# Novel electrochemical route to cleaner fuel dimethyl ether

**DOI:** 10.1038/s41598-017-07187-8

**Published:** 2017-07-31

**Authors:** Giuseppe Cassone, Fabio Pietrucci, Franz Saija, François Guyot, Jiri Sponer, Judit E. Sponer, A. Marco Saitta

**Affiliations:** 1 Institute of Biophysics, Czech Academy of Sciences, Královopolská 135, 61265 Brno, Czech Republic; 2 Sorbonne Universités, Université Pierre et Marie Curie Paris 06, Institut de Minéralogie, de Physique des Matériaux et de Cosmochimie, CNRS, Muséum national d’Histoire naturelle, Institut de Recherche pour le Développement, Unité Mixte de Recherche 7590, F-75005 Paris, France; 3 CNR-IPCF, Viale Ferdinando Stagno d’Alcontres 37, 98158 Messina, Italy

## Abstract

Methanol, the simplest alcohol, and dimethyl ether, the simplest ether, are central compounds in the search for alternative “green” combustion fuels. In fact, they are generally considered as the cornerstones of the envisaged “Methanol Economy” scenario, as they are able to efficiently produce energy in an environmentally friendly manner. However, despite a massive amount of research in this field, the synthesis of dimethyl ether from liquid methanol has never so far been reported. Here we present a computational study, based on *ab initio* Molecular Dynamics, which suggests a novel synthesis route to methanol dehydration – leading thus to the dimethyl ether synthesis – through the application of strong electric fields. Besides proving the impressive catalytic effects afforded by the field, our calculations indicate that the obtained dimethyl ether is stable and that it can be progressively accumulated thanks to the peculiar chemical pathways characterising the methanol reaction network under electric field. These results suggest that the experimental synthesis of dimethyl ether from liquid methanol could be achieved, possibly in the proximity of field emitter tips.

## Introduction

The last few years have witnessed a growing interest towards a “creative chemistry” of methanol (CH_3_OH), mainly because this compound is largely considered a very credible opportunity to produce “green” energy^1^. Not surprisingly, this is accompanied by massive investments aimed at the methanol industry, in China, Middle East, and South America, just to cite the leading investors. This interest has become extremely compelling because of the unsustainable levels of pollution reached in many countries and, on the other hand, thanks to the highly multifaceted, and well-established^1^, reaction network characterising the chemistry of methanol^2–7^. This compound represents indeed one of the feedstocks for the production of hydrocarbons^2, 5^, olefins^3^, di-hydrogen^4^, dimethyl ether (DME)^6^, and so on.

Among the reaction pathways that can originate from the simplest alcohol, formaldehyde (H_2_CO) and DME (CH_3_OCH_3_) synthesis hold a privileged place. Most of methanol is in fact converted to the simplest aldehyde (*i*.*e*., about 40%) with the aim of subsequently producing plastics, paints, plywood, as well as textiles. On the other hand, synthesis of DME, the simplest ether, presents an advantageous hub for the global energy production in the imminent future^1^. This compound has a cetane number of 55–60, sensibly higher than the 40–55 of common diesel fuel, and thus is a potential alternative fuel. In addition, DME is a non-toxic, non-corrosive, non-carcinogenic, and environmentally friendly substance, whose combustion exhaust gas is far cleaner than that of conventional diesel fuels^8^. Due to these evidences, it has been recently employed for internal combustion engines – along with common liquid petroleum gas (LPG)^8^. Even more interestingly, several leading multinational corporates such as Nissan, Volvo, and Isuzu, have already developed heavy vehicles with diesel engines running with DME.

The conversion of methanol to DME is however traditionally achieved at high temperatures (*i*.*e*., in the gas phase) and in presence of very specific catalysts^9–12^. Of course, the importance of liquid methanol resides *inter alia* in the fact that it is preferable over highly volatile and potentially explosive materials (*e*.*g*., di-hydrogen) for energy storage and transportation^8^. Thus, achieving a direct conversion of liquid methanol to DME could be a major breakthrough.

Electric fields can promote peculiar reaction channels and the first experimental evidence that an electric field can control chemical reactions has been very recently provided^13^. Aragones *et al*.^13^ have shown that a Diels-Alder reaction can be driven, enhanced, or even inhibited by the field strength and its polarity. It appears hence increasingly evident that in an imminent future oriented electric fields will be employed as smart reagents in chemistry^14^, with unthinkable implications for industry and society. On the other hand, recent *ab initio* molecular dynamics (AIMD) studies have succeeded in describing complex behaviour of molecular systems under strong electric fields in the case of water^15^, quantitatively confirmed by experiments^16–18^. In addition, external field strengths of the order of 0.50 V/Å have been also employed in order to reproduce *in silico* the historical Miller experiment^19^. By exploiting similar computational approaches, we have recently shown^20^ the feasibility of formaldehyde and methane synthesis through the application of electric fields to liquid methanol. Similarly, with an equivalent AIMD setup, methanol reactivity in proximity of electrified interfaces has been probed in order to characterise the first steps of the electro-oxidation of the simplest alcohol^21^.

Here we present a set of so far unreported chemical reactions which occur when a sample of pure liquid methanol is exposed to the effect of static and homogeneous electric fields, besides the mentioned observation of the one-step formation of formaldehyde and methane^20^. Anticipating our results, we observe that other relevant chemical species, such as DME and formaldehyde monohydrate, are formed at high field intensities. Moreover, DME appears to be a stable product which can be further accumulated by increasing the field strength. This way, a new reaction channel for the DME synthesis emerges from state-of-the-art AIMD computations that, along with the other reported reaction pathways, significantly extends the limits of the methanol reaction network.

## Results and Discussion

### Field-induced chemical reactions

Although the reaction network characterising the chemistry of methanol is highly multifaceted^2–7^, the industrially relevant reaction routes starting from the simplest alcohol are well-established^1^. However, the major reaction pathways that can be built by utilizing methanol as the primary reactant under the effect of an electric field are still unknown.

At relatively moderate field strengths (*i*.*e*., 0.30 V/Å) it is possible to induce the cleavage of the O-H covalent bond which results in the concept of generalization of *p* H in methanol^22^. This way, molecular dissociations are activated and protons H^+^ can migrate *via* a Grotthuss-like mechanism through the H-bond network. This process is thus assisted by a certain fraction of ionic species, such as the methanol cation CH_3_OH_2_^+^ (methyloxonium) and anion CH_3_O^−^ (methoxide) which are responsible for the ionic charge flow in the system and for enhanced contributions to the external electrostatic potential. The intensity of the local electric fields – due to the ions – can be of the order of 1 V/Å^23–25^, which strongly increases the overall molecular reactivity. For external electric field intensities stronger than 0.50 V/Å, we observe several field-induced chemical reactions. Indeed, a field strength of 0.55 V/Å is able to induce the formation of formaldehyde molecules, as well as of methane and water, according to the following reaction^20^

1 $$2C{H}_{3}OH\,\mathop{\longrightarrow }\limits^{{\rm{E}}}\,{H}_{2}CO+C{H}_{4}+{H}_{2}O\,.$$

Formaldehyde, being an extremely reactive compound, gives rise to a progressive complexification of the system. First, due to the water production, for field intensities stronger than 0.55 V/Å formaldehyde hydration takes place as in the following

2 $${H}_{2}CO+{H}_{2}O\,\mathop{\longrightarrow }\limits^{{\rm{E}}}\,C{H}_{2}{(OH)}_{2}\,,$$

and formaldehyde monohydrate is transiently observed.

More interestingly, at the same field strengths, methanol dehydration also occurs leading to the synthesis of DME according to:

3 $$2C{H}_{3}OH\,\mathop{\longrightarrow }\limits^{{\rm{E}}}\,C{H}_{3}OC{H}_{3}+{H}_{2}O.$$

**Figure 1:**
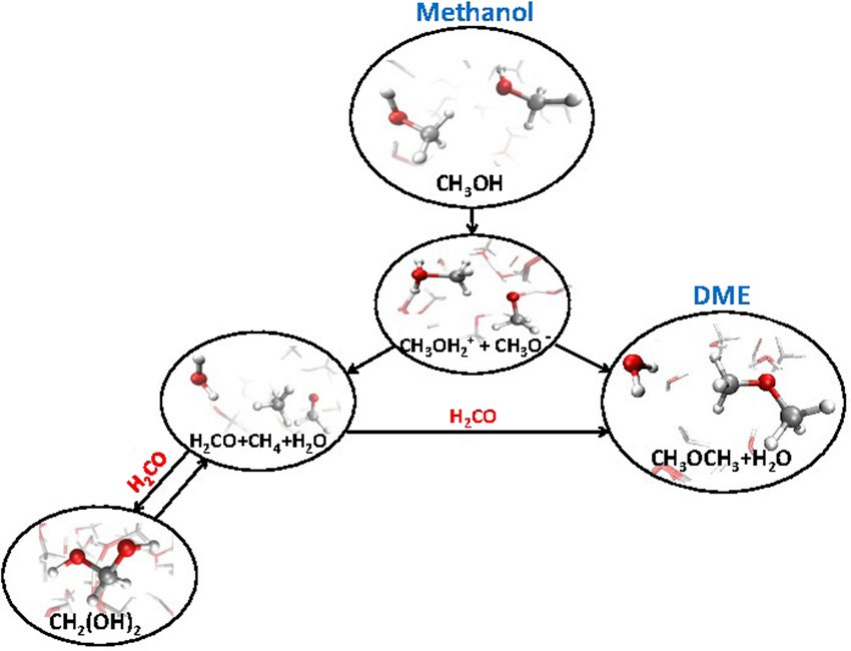
Methanol reaction network in presence of a static electric field. The application of this latter dissociates some methanol molecules (second panel from the top). Strengths above 0.50 V/Å are able to recombine the formed methanol cations and anions both into formaldehyde, methane, and water, both into DME and water (third panel from the top). Formaldehyde molecules will be further employed in order to synthesise also formaldehyde monohydrate (which has a strong tendency to dissociate) and DME.

**Figure 2:**
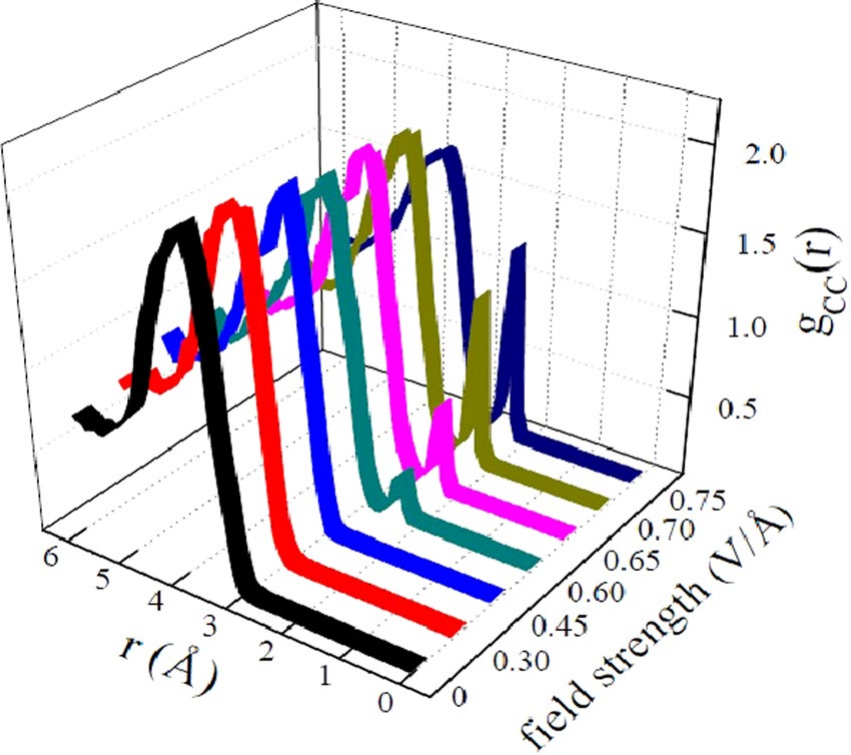
Carbon-carbon radial distribution function determined at different field strengths. Black curve: *E* = 0 V/Å; red curve: *E* = 0.30 V/Å; blue curve: *E* = 0.45 V/Å; cyan curve: *E* = 0.60 V/Å; pink curve: *E* = 0.65 V/Å; light green curve: *E* = 0.70 V/Å; dark blue curve: *E* = 0.75 V/Å. The synthesis of DME species starting from a field intensity of 0.60 V/Å is marked by the onset of a new peak located at ~2.4 Å. Above this strength, the stronger is the field the higher is the newly formed peak (*i*.*e*., a further increase of the field leads to a progressive accumulation of DME).

As shown in Fig. 1, the involved field intensities are able to open several reaction channels that connect the simplest alcohol to the simplest aldehyde on one hand and, on the other, to the simplest ether. The most direct chemical transformations that join methanol with formaldehyde (reaction (1)) and, separately, with DME (reaction (3)), involve the cation CH_3_OH_2_^+^ and the anion CH_3_O^−^ which act in both cases as intermediate species. In addition, the produced formaldehyde can either undergo hydration or yield DME, which represents the most abundant compound that has been synthesised in the system, as displayed in Table 1. Indeed, for field strengths of 0.60 V/Å and 0.65 V/Å, the transiently formed formaldehyde molecules have been readily employed for the synthesis of DME and formaldehyde monohydrate, yielding the molecular fractions shown in Table 1. For higher field strengths −0.70 V/Å and 0.75 V/Å – the system is temporarily characterised by an elevated degree of mixing. However, after few ps of dynamics performed at these field intensities, only water, DME, and methane species – in order of decreasing amount – are observed. Instead, formaldehyde, the most reactive species among the neutral ones, ultimately contributes to the process of molecular complexification of the system. In the end, DME is the most abundant (non-aqueous) synthesised species which has been accumulated along the simulation. This is clearly shown, besides the direct inspection of the trajectories, in Fig. 2, by the onset of a new peak at shorter distances (*i*.*e*., ~2.4 Å) in the carbon-carbon radial distribution function for fields exceeding 0.55 V/Å. This corresponds to the typical carbon-carbon intramolecular distance found in DME. Moreover, the peak gets more and more pronounced by further increasing the field strength, indicating the progressive accumulation of the simplest ether. Incidentally, as shown in Fig. 1, the basin of DME in the field-induced reaction network of methanol is the only one that can be reached by following two different chemical pathways, and the only one displaying exclusively incoming “chemical fluxes” (*i*.*e*., DME is chemically inert). In addition, the enthalpy gain associated with the formation of DME rather than formaldehyde (shown in Fig. S1 of the Supplementary Information (SI)) renders, at high field intensities, the simplest ether synthesis a sort of chemical sink. In fact, although also formaldehyde, methane, and water can be formed from the methanol counterions^20^, DME and water synthesis is thermodynamically privileged for high field intensities, as shown in Fig. S1 of the SI. This way, DME is progressively accumulated.

**Table 1:** Total populations (percent fractions) of the molecular species present in the system at the end of each simulation carried out at field strengths of 0.55 V/Å, 0.65 V/Å, and 0.75 V/Å.

**Molecular species**	0.55 V/**Å**	0.65 V/**Å**	0.75 V/**Å**
Formaldehyde	1 (3%)	0	0
Methane	1 (3%)	2 (6%)	2 (6%)
Water	1 (3%)	3 (9%)	6 (19%)
Formaldehyde monohydrate	0	1 (3%)	0
Dimethyl ether (DME)	0	2 (6%)	4 (13%)

The fractions are determined as the ratio between the number of molecules of a given species and the number of total methanol molecules composing the original sample (*i*.*e*., 32). The lack of formaldehyde above the field threshold which is able to create it (*i*.*e*., 0.55 V/Å) is due to its extensive employment in diverse chemical reactions leading both to formaldehyde hydration both to DME synthesis. Formaldehyde acts as a reactive intermediate species which contributes to the complexification of the system.

The energy contributions stemming from field strengths of those orders of magnitude are about 30–35 kcal/mol^20^. Although this estimate has been obtained by means of accurate state-of-the-art *ab initio* and metadynamics techniques^26^, a less sophisticated calculation shown in Fig. S2 of the SI indicates values within the same range. Albeit field strengths of the order of 0.50–0.75 V/Å are of course of extreme intensity from the macroscopic point of view, it is nowadays well established that field intensities of (or greater than) 1 V/Å are locally found at the microscopic scale in disparate condensed systems in presence of simple solvated ionic species^23–25^, or even at the surface of clean apolar oxides^27^. In field emitter tips experiments, field strengths of 1–3 V/Å are recorded^28–30^ and it has been proven that intensities of 0.30 V/Å are necessary in order to induce water dissociation^16–18^ and, in general, to significantly shift the bonding electrons^14^, thus confirming the predictions by some of us^15^, achieved with a very similar computational approach. Very recently, fields within this order of magnitude have been again experimentally detected at the tip proximity^31^. All these evidences strongly suggest the experimental feasibility of the proposed reactions, by exploiting the high field capability of, *e*.*g*., field emitter tips.

### Dimethyl ether synthesis – The important role of solvation

**Figure 3:**
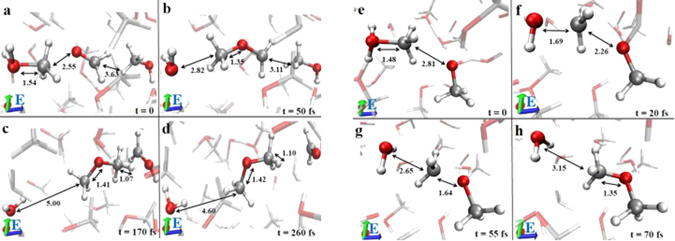
The least (**a**–**d**) and the most (**e**–**h**) favourable DME formation mechanisms in presence of a static electric field oriented along the positive *z*-axis (*i*.*e*., the blue cartesian axis direction) at a strength of 0.60 V/Å. Red, silver, and white coloring refers to oxygen, carbon, and hydrogen atoms, respectively. Several distances (shown in Å) have been chosen in order to better follow the reactions. (**a**–**d**) Starting from a methanol cation, a formaldehyde and a nearby methanol molecules (**a**), a water molecule and a DME cation are formed by transferring a methenium CH_3_^+^ (**b**). In 120 fs the DME cation is neutralized thanks to an hydride H^−^ stemming from the nearby methanol’s methyl group (**c**). Deprotonation of this latter species leads to the formation of another formaldehyde molecule along with DME and water (**d**). (**e**–**h**) A proton transfer event (**e**), assisted by the presence of a nearby methoxide anion, leads to the cleavage of the C-O bond of the newly formed CH_3_OH_2_^+^ cation (**f**) (*i*.*e*., the bond length evolves from 1.48 Å to 1.69 Å in 20 fs). Local electrostatic effects produce thus the fast release of the methenium ion CH_3_^+^ that, just after the “umbrella inversion” (**g**), recombines with the methoxide leading to the formation of DME and to the release of water (**h**).

As shown above, field strengths of 0.60 V/Å and higher lead to the synthesis of DME. Figure 3 displays the two microscopic mechanisms that are able to give rise to DME in the simulation sample. As a first consequence of the application of an electric field, some molecules/ions tend to align their least electronegative atomic sites towards the field direction (see Fig. 6 of ref. 22). However, this condition is not strictly fulfilled in a highly structured system such as a liquid where intermolecular interactions may outweigh the external field contribution. Additionally, the field-activated proton transfer in the system makes possible ionic configurations such as those shown in Fig. 3a,e for the methanol cation CH_3_OH_2_^+^, this latter having its own positive pole pointed in the opposite direction with respect to the field orientation. Note that the orientation of CH_3_OH_2_^+^ with respect to formaldehyde in this configuration hampers a direct proton transfer from the former’s oxygen to the latter molecule. The concerted action of the aldehyde and of the external field, along with the surrounding presence of other ionic species, leads to the cleavage of the C-O bond of the methanol cation. A methenium CH_3_^+^ is thus released, forming a DME cation by combining with formaldehyde, while a water molecule is released (Fig. 3b). The newly formed cation rapidly receives an hydride H^−^ from a neighbour methanol evolving thus into DME (Fig. 3c). The hydride stems from the methyl group of a nearby methanol molecule, which evolves into formaldehyde (Fig. 3d) after the release of a proton in favour of the solvent. Overall, the mechanism implies the destruction of one formaldehyde molecule and the creation of a new one, so that this species can be seen as a catalyst.

There exists also another, simpler (one-step) and faster, reaction channel by which DME can be synthesised (Fig. 3e,h), once more assisted by local frustrations. Indeed, as shown in Fig. 3e, neither methoxide – which has its dipolar axis perpendicular to the field direction – nor methyloxonium – which, as the result of a proton transfer, is oriented with the excess proton in opposition to the field – are arranged as expected for free dipoles in an electrostatic field. In such a configuration, the neutralization process takes place as schematically depicted in Fig. 3e,h and as confirmed by the identification of the Wannier charge centers, shown in Fig. S3 of the SI, which allows for the localization (and hence visualization) of the electron pairs^32^. The C–O bond of CH_3_OH_2_^+^, already sizably longer than in methanol because of the extra proton, is further weakened by the presence of the nearby methoxide anion (Fig. 3f), and gets broken within a few dozens of fs. The released methenium ion CH_3_^+^, featuring an “umbrella inversion”, approaches the oxygen atom of CH_3_O^−^ (Fig. 3f,g). The process leads to the formation of DME coupled with a release of water (Fig. 3h).

These two mechanisms thus show that the local environment plays a major role in assisting the chemical reactions by acting *inter alia* as a sort of *reservoir* of proton H^+^ and hydride H^−^ acceptor/donor sites. Of course, even series of combined synthesis processes have been recorded, as shown in Fig. S4 of the SI.

**Figure 4:**
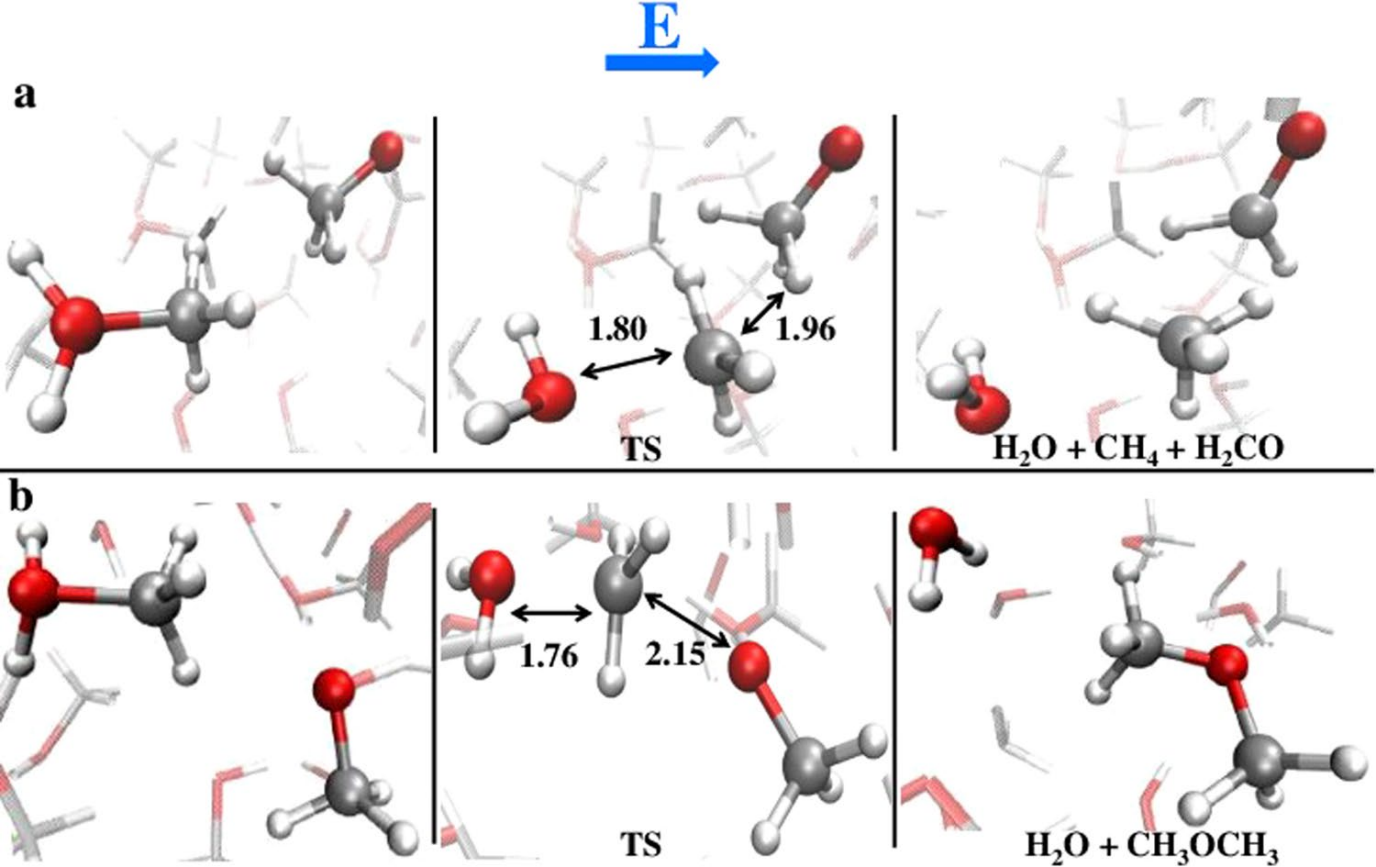
A twofold reaction channel. Depending on the mutual orientation of methoxide CH_3_O^−^ and methyloxonium CH_3_OH_2_^+^, the reaction proceeds yielding either formaldehyde, methane, and water (**a**) or DME and water (**b**). In the central panels, the transition states of both reactions, obtained by means of a committor analysis performed at $300$ K and in presence of field strengths of $0.55$ V/Å (**a**) and $0.60$ V/Å (**b**), are shown.

As laid out, the main intermediate state of the DME synthesis and the basic mechanism of formation of formaldehyde are characterised by the presence of the two counterions of methanol (Fig. 1). However, it is clear that two very different reaction channels can be undertaken by the system depending on the mutual orientation of nearby methyloxonium and methoxide ions. At intense field strengths, the head-to-tail arrangement ^+^H_2_O–CH_3_…^−^O–CH_3_ leads to DME and water formation (*i*.*e*., reaction (3), Fig. 3e,h). On the other hand, if the adjacent parts of the methanol counterions are the respective methyl groups, the reaction evolves towards the synthesis of formaldehyde, methane, and water (*i*.*e*., reaction (1))^20^. These two reaction channels are shown in Fig. 4. The transition states of both reactions have been identified by means of a committor analysis^33^ and are depicted in the central panels of Fig. 4. By definition, the identification of a transition state splitting the probability to reach reactants or products implies the existence of a residual free energy barrier even under the intense electric field triggering the reactions. It is known, however, that condensed phase reactions involving ionic states (*e*.*g*., proton transfer) may be described in terms of dynamical barriers, that are modulated in time by the fluctuations of the environment-induced electrostatic field^34, 35^.

Since the observed reaction mechanisms are intimately related to the multifaceted and collective character of the medium, it is to be expected that the chemical pathways undertaken by the system in condensed phase are entirely different from any reproduced gas phase reactions. Indeed, by performing additional simulations starting from the ionic intermediate configurations shown in the left panels of Fig. 4 but placed in the gas phase – *i*.*e*., by removing the solvent –, we observed that both reactions (1) and (3) are barrierless and spontaneously proceed even in absence of any electrical perturbation (see the SI for details).

**Figure 5:**
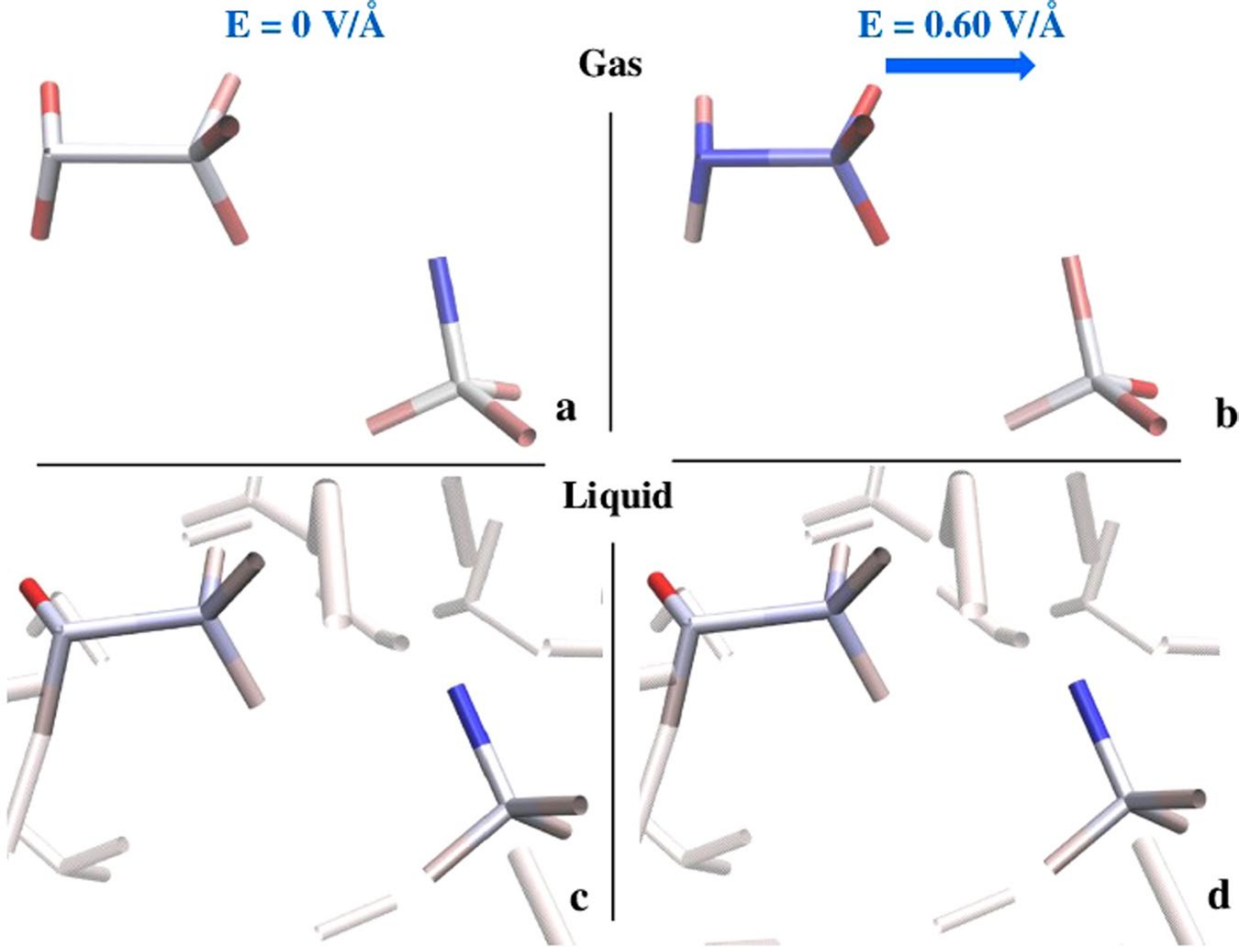
Löwdin population analysis of CH_3_OH_2_^+^ and CH_3_O^−^ in the gas phase (**a**,**b**) and in the liquid phase (**c**,**d**) without (**a**,**c**) and with an external electric field at a strength of $0.60$ V/Å (**b**,**d**). The blue arrow shows the field orientation. The coloring method follows the atomic electron populations shown in Table S1. In particular, the atomic population values relative to the expected ideal atomic valence population (*i*.*e*., $6$ for the oxygen, $4$ for the carbon, and $1$ for the hydrogen atoms) are plotted. Deep blue coloration indicates a strong positive value with respect to the expected ideal valence population; deep red coloration illustrates a strong negative value with respect to the expected valence population; white coloration stems from estimated values very close to the standard ideal electronic population of each atomic species.

A useful method for the evaluation of local polarization effects is the electron population analysis, which allows for the estimate of the partial number of valence electrons on each atomic site. Albeit the absolute values are somewhat dependent on the choice of the wavefunctions basis set, relative estimates under different external conditions may carry fundamental insights. Hence, in order to quantify the solvent effects in altering the bare action of the applied field on the reactant atoms of the most important reaction (*i*.*e*., reaction (3)), a Löwdin population analysis^36^ has been performed. Four cases have been taken into account: two of them refer to the counterions in absence of the field, both in the gas phase and in explicit solvation (Fig. 5a,c), whereas the same two systems have been also considered in the presence of the field that induces the reaction yielding DME (Fig. 5b,d). The colors of the molecules shown in Fig. 5 underline the fact that the external field is strongly screened by the solvent. The valence electron populations present on each atomic species of the reactant ions are listed in Table S1 of the SI. In presence of an external field the reactants show large variations of the valence atomic charges when evaluated in the gas phase: the oxygen atom of the methoxide anion shows a considerable lower electron population than in absence of the field. At the same time carbon, oxygen, and the non-methyl hydrogen atoms of CH_3_OH_2_^+^ display larger electron populations with respect to the zero-field case (see Table S1). Due to the field action, a partial negative charge has been thus transferred from the anion to the cation, as visible in Fig. 5-a/b (see also Fig. S5 of the SI for the evaluation of the respective Wannier centers). By explicitly taking into account the solvent effects, the situation dramatically changes. The results, described in Table S1 and better visualized in Fig. 5, show indeed that the field-induced effects manifested in the gas phase (Fig. 5a,b) are no longer detectable in the liquid (Fig. 5c,d); all the atomic electron populations of the two ions are almost independent of the application of the field, suggesting that local solvent contributions are sizable. If, on one hand, the local solvation environment hampers highly polarized – and thus reactive – molecular states, on the other hand it plays a key role in shaping the final steps of a reaction, orienting it towards specific channels and hence products, as also shown in Fig. 4.

Finally, the mean lifetime of formaldehyde – the most reactive neutral species in our sample – appears to be rather independent of the field strength (Table S2 of the SI). At high intensities the local environment is indeed decisive on the reactivity of the simplest aldehyde. Hence, the field plays a crucial role as it affects the local ionic concentration of a solution and, as it is well-known, it can create and stabilize ionic transition states^14^. Moreover, as shown in the central panels of Fig. 4, the transition states characterising the main reactions here presented are resemblant to the reactants configurations, proving the decisive energetic contribution carried by the application of the field (*i*.*e*., 30–35 kcal/mol^20^) and its own natural consequences (*i*.*e*., molecular ionizations). Nevertheless, the fate of a given reaction is dictated by local environment circumstances. In fact, once strong local electrostatic contributions are generated by the field action, molecular correlations are decisive in pushing a given intermediate state into a specific reaction product.

## Conclusions

Using *ab initio* Molecular Dynamics we investigated the effects of the application of intense static electric fields to bulk liquid methanol. Our results reveal that the reaction network characterising the fundamental chemistry of the simplest alcohol can be significantly extended by exploiting the catalytic effects of the electric fields. Among the possible observed reaction pathways, the most relevant synthesised species is the simplest ether, dimethyl ether (DME), which is a renowned “green” fuel actually employed both in some Diesel engines and for internal combustion engines.

We demonstrate that, if the electric field plays a crucial role in initiating those chemical processes, local molecular correlations are decisive in routing the system towards a peculiar reaction pathway. The combined action of the field and of the intermolecular interactions leads to a multifaceted chemistry where, besides DME, products such as formaldehyde, methane, and formaldehyde monohydrate are also produced. Although the main reaction channels of synthesis of the simplest aldehyde and that of the simplest ether are very similar, the strong difference in molecular reactivity between formaldehyde and DME leads to asymmetric chemistries shown by these two compounds. On one hand, formaldehyde is employed as an extremely reactive precursor of all the other synthesised species whereas, on the other, DME is fully stable and it represents a sort of “sink” of the field-induced methanol reaction network, where all the observed chemical transformations seemingly converge. Our findings are quite promising, as they explicitly call out for experimental feasibility of synthesis and accumulation of DME from liquid methanol by means of suitable devices, including for instance high field emitter tips.

## Methods

We used the software package Quantum ESPRESSO^37^, based on the Car-Parrinello (CP) approach^38^, to perform AIMD simulations of a sample of methanol molecules under the action of intense electric fields applied along a given direction (corresponding to the $z$-axis). The implementation of an external electric field in numerical codes based on density functional theory (DFT) can be achieved by exploiting the modern theory of polarization and Berry’s phases^39^ (see, *e*.*g*., ref. 40). Our sample contained $32$ CH_3_OH molecules (*i*.*e*., $192$ atoms) arranged in a cubic cell with side parameter $a=12.93$ Å, so as to reproduce the experimental density of $0.79$ g/cm^3^ at about $300$ K. As usual, the structure was replicated in space by using periodic boundary conditions.

The *ab initio* simulations have been carried out at the average temperature of $300$ K after an equilibration run of 5 ns performed by means of the typical Optimized Potential for Liquid Simulations (OPLSs) (see, *e*.*g*., ref. 41) in order to prepare a suitable initial atomic configuration for AIMD. In the latter we gradually increased the intensity of the electric field with a step increment of about $0.05$ V/Å from zero up to a maximum of $0.75$ V/Å. In the zero-field case we performed a dynamics of $6$ ps whereas, for each other value of the field intensity, we ran the dynamics for $3$ ps, thus cumulating a total simulation time longer than $50$ ps. Additionally, once all the chemical reactions leading to the production of formaldehyde, methane, water, DME, and formaldehyde monohydrate occurred, a stability analysis of the formed compounds was carried out. Indeed, in a series of independent CP molecular dynamics simulations, the electric field has been switched off and the dynamics have been followed for times longer than $20$ ps. It turns out that all the formed species remain stable under the field shutdown. As far as the effects generated by low-to-moderate field intensities on liquid methanol are concerned, the interested reader can refer to ref. 22 for extensive analyses on a “baseline” simulation. In addition, several tests have been conducted in order to better clarify the role played by the methanol counterions. To this aim, we ran AIMD simulations both on numerical samples containing methanol counterions placed in methanol “by hand” in absence of the field, both in samples where – once formed a certain fraction of ionic species – the external field has been abruptly switched off (*i*.*e*., instantaneously all the molecules were in a highly polarized state). It turned out that, in both cases, the absence of the field leads to a global neutralization process of the ions by means of cooperative proton transfers across the H-bonded network.

The fictitious electronic mass was set to a value of $300$ a.u., with a cutoff energy of $35$ Ry for the plane-wave representation of wavefunctions and of $280$ Ry for the charge density, with a timestep of $0.12$ fs. With such cutoff values the sample is described in a reliable way since the core electronic interaction is being depicted through ultrasoft pseudopotentials (USPP) generated *via* the Rappe-Rabe-Kaxiras-Joannopoulos (RRKJ) method^42^. As an approximation of the exchange and correlation functional, we adopted the Perdew-Burke-Ernzerhof (PBE) functional^43^, which belongs to the generalized gradient approximation (GGA). The dynamics of ions was simulated classically within a constant number, volume, and temperature (NVT) ensemble, using the Verlet algorithm and a Nosé-Hoover thermostat set at a frequency of $13.5$ THz.

In order to characterise the transition states of the reactions leading on one hand to formaldehyde, methane, and water and, on the other, to DME and water at their own field thresholds, a committor analysis^33^ has been performed. By choosing dozens of structures that were considered as plausible candidates for being the transition state of each simulation, $50$ unbiased trajectories of $200$ fs each have been performed starting from each candidate and differing by the initial random velocities extracted from a Maxwell-Boltzmann distribution at 300 K. We have identified a configuration as belonging to the transition state ensemble when it is committed to the reactants or products basin with a probability of 50 ± 10%.

Löwdin population analysis has been performed by projecting the wavefunctions onto their standard (pseudo) atomic basis sets. By using a simple Gaussian broadening with spread equal to $0.0002$ Ry, the projected density of states and the Löwdin populations have been thus evaluated. In addition, with the aim of checking the reliability of the electron population analysis and tracing the bonds behaviour of the main chemical reaction here presented, also the Maximally Localized Wannier Functions (MLWF)^32^ as well as their charge centers have been determined (see Figs S3 and S5 of the SI). Finally, by considering the gas phase counterparts of the chemical reactions leading on one hand to formaldehyde, methane, and water and, on the other, to DME and water, the intermolecular interactions between the local reactants have been evaluated through the Noncovalent Interactions (NCI) scheme^44^, as shown in Fig. S6 of the SI. See the SI for further details and for additional analysis.

### Data Availability

All the data are freely and promptly available to readers.

## Electronic supplementary material

Supplementary Information

Supp Video
