# Supplementary material for: Novel electrochemical route to cleaner fuel dimethyl ether
Source: Sci Rep. 2017 Jul 31;7:6901. doi: 10.1038/s41598-017-07187-8 (PMC5537294; doi:10.1038/s41598-017-07187-8)
Supplement: Supplementary file 1 — Supplementary Information [file 41598_2017_7187_MOESM1_ESM.pdf]

Supplementary Information

Novel electrochemical route to cleaner fuel dimethyl ether

Giuseppe Cassone[1a], Fabio Pietrucci[2b], Franz Saija[3c],

François Guyot[2d], Jiri Sponer[1e], Judit E. Sponer[1f], A. Marco Saitta[2g]

[1] *Institute of Biophysics - Czech Academy of Sciences,*

Královopolská 135, 61265 Brno, Czech Republic

[2] *Sorbonne Universités,*

Université Pierre et Marie Curie Paris 06,

Institut de Minéralogie,

de Physique des Matériaux et de Cosmochimie,

CNRS, Muséum national d'Histoire naturelle,

Institut de Recherche pour le Développement,

Unité Mixte de Recherche 7590,

F-75005 Paris, France

[3] *CNR-IPCF, Viale Ferdinando Stagno d'Alcontres 37,*

98158 Messina, Italy

(Dated: May 30, 2017)

[a] Email: cassone@ibp.cz

[b] Email: fabio.pietrucci@impmc.upmc.fr

[c] Email: saija@ipcf.cnr.it

[d] Email: fguyot@mnhn.fr

[e] Email: sponer@ncbr.muni.cz

[f] Email: judit@ncbr.muni.cz

[g] Email: marco.saitta@impmc.upmc.fr

With the aim to partially understand the physical reasons behind the observation that DME molecules can be regarded as a sort of “sink” or “chemical well” of the methanol reaction network under electric field, a systematic investigation of the enthalpy of the system has been carried out. In standard *ab initio* Molecular Dynamics (AIMD) simulations this latter quantity is determined as $H = E(KS) + P \cdot V$, where E(KS) is the Kohn-Sham total energy of the system, P is the pressure, and V is the volume which holds a fixed value within our NVT simulation. In particular, the total enthalpy of the system has been sampled for each event of formation of a DME molecule (in symbiosis, of course, with the release of a water molecule; *i.e.*, reaction (3) of the main text). Moreover, the same calculation has been performed for the synthesis of formaldehyde (along with methane and water; *i.e.*, reaction (1) of the main text). The sampling process has been conducted for dynamics of the order of 1 ps subsequent to a given formation event. The time-scale, or better the temporal cutoff for the accumulation of data, is clearly dependent on the eventual occurrence of other chemical transformations in the numerical sample and, for the formaldehyde case, it depends also on the occurrence that the simplest aldehyde undergoes to further reactions. In such a way, the variation of the total enthalpy of the system can be entirely ascribed either to the formation of DME or to the formaldehyde synthesis. Therefore, the accumulation of the statistical points has been carried out for three different field strengths: 0.60 V/Å, 0.65 V/Å, and 0.70 V/Å. The results, shown in Fig. S1, indicate that the formation of DME molecules

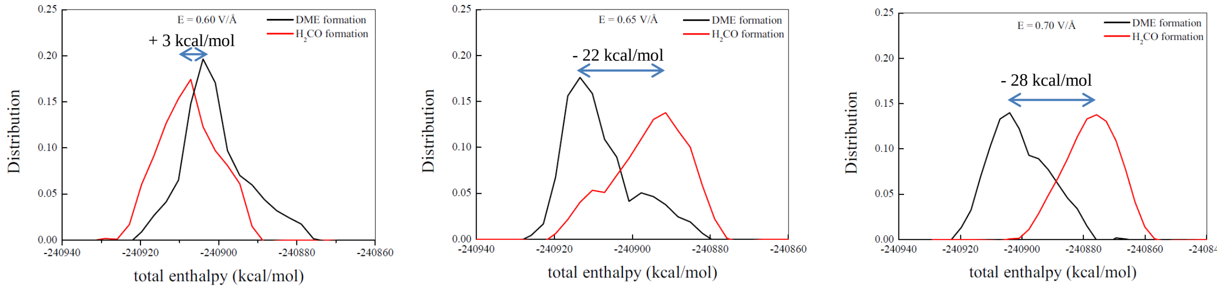

FIG. 1. Distributions of the total enthalpy of the whole system (*i.e.*, composed by 192 atoms) ascribable to the DME (black curves) and to the formaldehyde (red curves) formation. Although at a field strength of 0.60 V/Å (left) the creation of formaldehyde is very slightly favoured with respect to that of DME (*i.e.*, by 3 kcal/mol), at higher field intensities the average enthalpy of the system associated with the DME synthesis is 22 kcal/mol and 28 kcal/mol, at 0.65 V/Å (center) and at 0.70 V/Å (right), respectively, lower than that characterising the formaldehyde formation.

becomes progressively more favoured with respect to that of the simplest aldehyde as the field intensity is increased. In fact, the difference in the average enthalpy of the system

associated either to the formation of DME or to that of formaldehyde gets more pronounced with the field strength. This way, the synthesis of DME will be preferred, from the energetic side, at those regimes. Finally, once formed, DME molecules do not undergo to any further chemical transformations, leading to the progressive accumulation laid out in the main text.

A rough analysis of the energetic contribution carried by the applied electric field has been carried out. In particular, by taking as the reaction coordinate the sequence of molecular configurations characterising the most prominent reaction mechanism leading to the synthesis of DME (see Fig. 3-e-h of the main text), a series of self-consistent field (*i.e.*, single point) calculations in absence of the electrical perturbation has been performed. In Fig. S2 the energy profile, reproduced by slightly smoothing the single point energy of each molecular state describing the brief dynamical trajectory of the progress of the reaction under investigation, is thus shown. It is evident that an energy barrier of about $30 - 35$ kcal/mol separates the instantaneous intermediate (I) to the products (P) state. This value is fully ascribable to the field action which renders almost barrierless the chemical reaction at a strength of 0.60 V/Å. A very powerful method that allow for the evaluation of the free-energy landscape of a given process [1] has very recently demonstrated that the energetic contribution of the electric field in promoting the synthesis of formaldehyde, methane, and water in liquid methanol falls in the same range of values [2]. Incidentally, this latter reaction shares a very similar intermediate state with the investigated reaction leading to the DME and water synthesis, giving *a posteriori* more reliability to the present calculation shown in Fig. S2.

In order to characterise the electronic ground state properties of the investigated system, we exploited the Maximally Localized Wannier Functions (MLWF) [3, 4]. One of the key factors that can be extracted from the MLWF are their charge centers which are a sort of quantum equivalent of the classical concept of the location of an electron pair and thus allow for a direct visualization of the bonds behaviour. The Wannier charge centers characterising the dynamical progress of the reaction leading to the DME synthesis at 0.60 V/Å are displayed in Fig. S3. In particular, Fig. S3-a shows a just formed methyloxonium cation (left) and a methoxide anion (right). As expected, the charge centers identifying both C-O bonds are slightly shifted towards the oxygen atoms of the shown species. In Fig. S3-b, it can be appreciated the cleavage of the methyloxonium C-O bond and the further approach of the respective Wannier center to the oxygen atom of the forming water molecule. This leads to the release of the methenium cation CH_3^+ as a transient species. As a direct consequence of

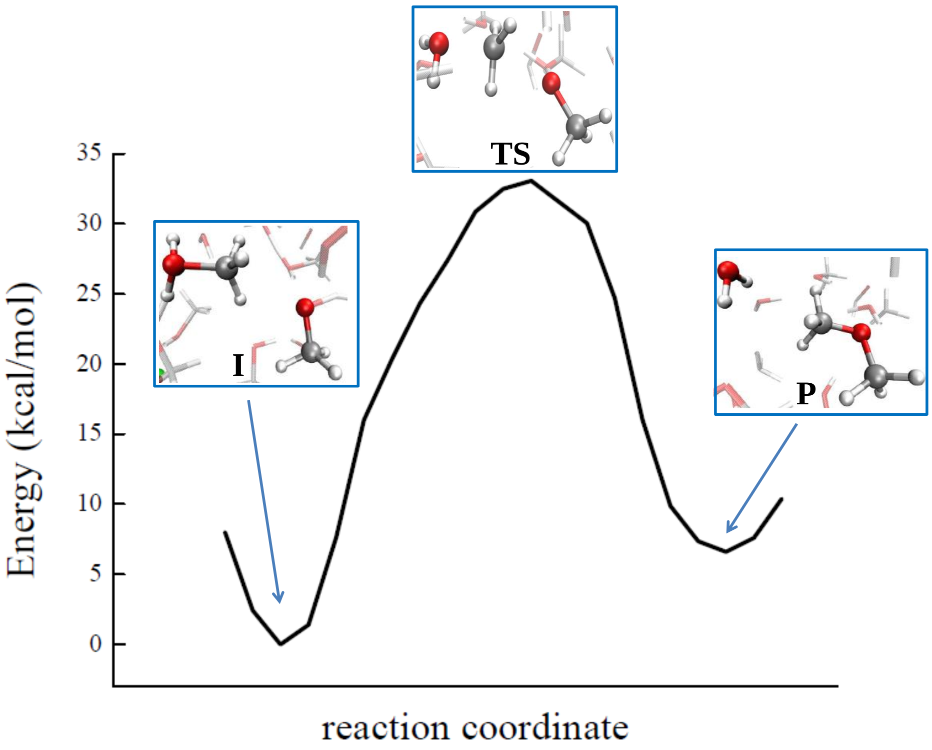

FIG. 2. Smoothed energy profile at zero-field obtained by determining the ground state energy of the molecular configurations characterising the dynamical evolution of the major reaction mechanism leading to the synthesis of DME and water. In particular, 15 different snapshots extracted from one of the original trajectories describing the reaction have been sampled. The range of configurations here considered goes from the intermediate state (I) to the products state (P) and passes through the evaluated transition state (TS). An idealized reaction barrier – in presence of the explicitly treated "solvent" – of about 33 kcal/mol has been recorded.

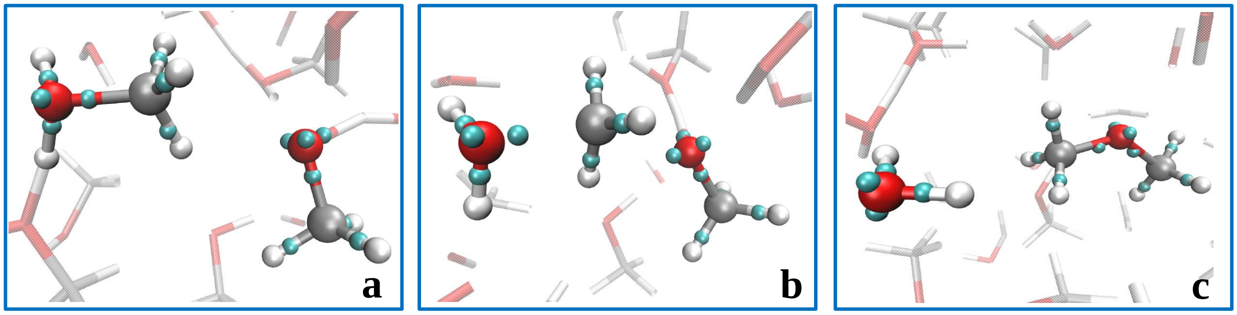

FIG. 3. Wannier charge centers (cyan small spheres) characterising the dynamical evolution of the main reaction channel that leads to the simultaneous formation of DME and water for a field strength of 0.60 V/Å.

the peculiar recombination process, a DME and a water molecule are synthesised and their respective Wannier centers are rapidly stabilized (Fig. S3-c)

As laid out in the main text, the electric field is able to induce a peculiar cooperativeness between the molecules. These effects are particularly colorful when the application of the

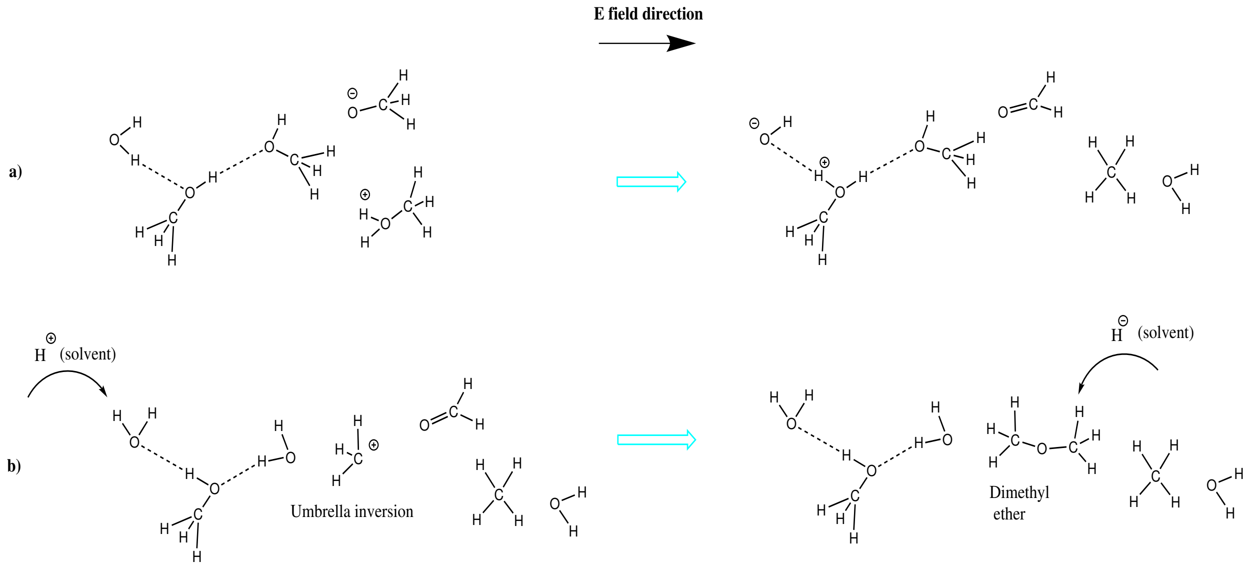

FIG. 4. Concerted cooperative molecular mechanisms occurring at 0.70 V/Å that allows for the onset of a formaldehyde in the sample (a). However, this species will be employed for the formation of a DME molecule (b). These sketches show the key role played by the solvent in assisting the chemical reactions and represent a striking combination of the synthesis processes of formaldehyde, on one hand, and DME, on the other. The overall process occurs in about 300 fs.

electrical perturbation is performed in a highly correlated system such a liquid. This way, at intense field strengths, concerted reaction mechanisms are recorded, as shown in Fig. S4, where an impressive sequence of synthesis processes are highlighted. Here (Fig. S4-a), a newly formed formaldehyde through the standard process – shown also in the upper panels of Fig. 4 of the main text – interacts with a just created methanol cation (Fig. S4-b) leading to the onset of DME (Fig. S4-b). This scenario shows again that the local environment plays a major role in assisting the chemical reactions by acting *inter alia* as a sort of *reservoir* of proton H^+ and hydride H^- acceptor/donor sites.

In the main text we have shown how the solvent is able to locally screen the field-induced polarization effects that would be strongly manifested in an hypothetical gas phase. To this aim, a Löwdin population analysis [5] has been performed and the coloring of the atomic sites of the main intermediate configuration leading to the synthesis of DME (Fig. 5 of the main text) stems from Table S1. This kind of analysis has been conducted both by explicitly taking into account the solvent contribution both by removing the solvent and considering an idealized gas phase counterpart of the reactants counterions that, by recombining, are able to give rise to DME and water. In order to additionally check the current population analysis, the Wannier centers stemming from the MLWF [3, 4] have been evaluated for the

	**E=0 V/Å**				**E=0.60 V/Å**			
**Atom**	**$CH_3OH_2{}^+$**		**CH_3O^-**		**$CH_3OH_2{}^+$**		**CH_3O^-**	
	**Gas**	**Liq**	**Gas**	**Liq**	**Gas**	**Liq**	**Gas**	**Liq**
H_1	0.65	0.61			0.81	0.62		
H_2	0.66	0.66			0.91	0.66		
O	6.25	6.26	6.61	6.52	6.43	6.29	5.87	6.54
C	4.25	4.24	4.18	4.22	4.32	4.25	4.15	4.22
H_3	0.80	0.83	0.87	0.84	0.79	0.82	0.80	0.81
H_4	0.78	0.77	0.86	0.85	0.77	0.77	0.81	0.84
H_5	0.77	0.81	0.89	0.87	0.76	0.79	0.77	0.89

TABLE I. Löwdin atomic valence electron populations of the two reactants counterions (*i.e.*, methyloxonium and methoxide), in the gas (Gas) and in the liquid (Liq) phases both in presence and in absence of an external electric field. The rows corresponding to oxygen and carbon atoms have been highlighted since, in principle, the eventual field-induced polarization effects are more evident on these atomic sites. $H_{1,2}$ sites refer to the "alcoholic" hydrogen atoms of $CH_3OH_2{}^+$ whereas $H_{3,4,5}$ represent the methyl hydrogen atoms of the latter and of CH_3O^-.

same four cases. It turns out that whereas the difference in the centers locations between the "solvated" intermediate states in absence and in presence of the field is in practice negligible (*i.e.*, they are stackable and the result in presence of a field strength of 0.60 V/Å is shown in Fig. S3-a), in the gas phase the field induces a visible shift of the charge as shown in Fig. S5. Thus, though in the framework of the MLWF an over-localization of the charges is present by construction, the field-induced shift of the charge appears manifest in the gas phase at these strengths.

Because of the lack of traces of formaldehyde at the end of our numerical experiment (*i.e.*, at 0.75 V/Å), the mean lifetime of this species has been evaluated at different electric field strengths, as shown in Table S2. It turns out that do not exist an analytical relationship – at least within the explored range of field intensities – between these two quantities and, from the analysis of the trajectories, it appears clear that at high strengths the local environment (*i.e.*, the "solvent") is much more decisive on the reactivity of a given species than the field intensity.

In addition to these evidences, another aspect that suggests the key role played by the "solvent" is represented by the fact that the transition states shown in the central panel of Fig. 4 of the main text – determined through a committor analysis [6] at our numerical

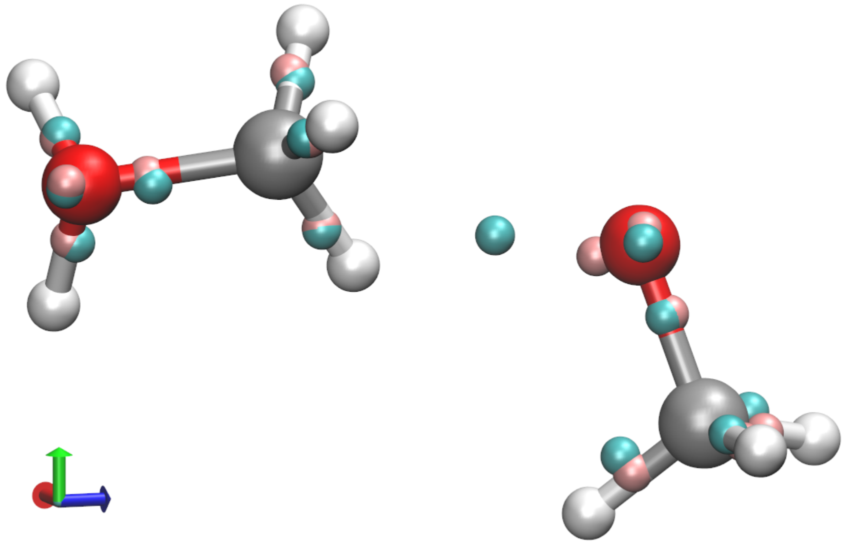

FIG. 5. Wannier charge centers in absence (pink small spheres) and in presence (cyan small spheres) of a field strength of 0.60 V/Å characterising the two reactants counterions configuration in the gas phase (*i.e.*, without the screening induced by the "solvent"). In the region separating the two counterions, a manifest field-induced shift – against the field direction – of the electronic charge occurs. The field direction is parallel to the z-axis which coincides with the blue cartesian axis of the reference system shown at the bottom.

**Field strength (V/Å)**	τ **(ps)**
0.55	1.1
0.60	0.1
0.65	1.6
0.70	0.7
0.75	*n.a.*

TABLE II. Formaldehyde mean lifetimes (second data column) at different field intensities (first data column).

experiment conditions – are no longer the same in the gas phase, as expected. Moreover, by performing AIMD simulations starting from the intermediate reactants configurations of the synthesis of formaldehyde and methane, on one side, and of DME, on the other, (see the left panels of Fig. 4 of the main text) without the solvent contribution (*i.e.*, in an idealized gas phase), we observe that both reactions proceed spontaneously even in absence of an external electrostatic gradient. This evidence led us to perform an additional analysis. By considering thus two ideal isolated gas phase systems composed by $CH_3OH_2{}^+$ and CH_3O^- without any

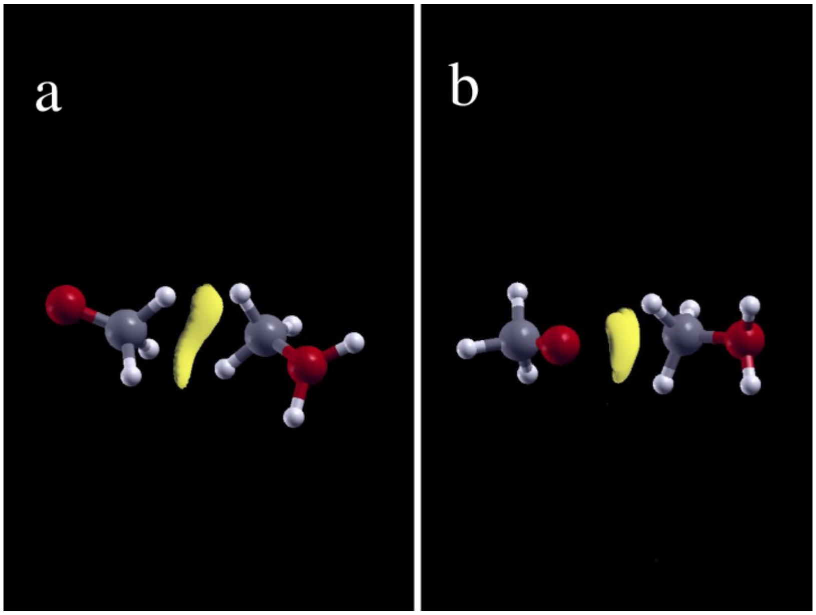

FIG. 6. NCI between the two local reactants (considered in the gas phase) just before the formation of formaldehyde, methane, and water (a) and DME and water (b) in a collateral numerical experiment. The isovalue chosen in order to visualize the NCI surface is 0.4 but any value between 0.3 and 0.6 allow for a correct visualization of them.

electrical perturbation, the Noncovalent Interactions (NCI) [7] have been evaluated. As shown in Fig. S6, the intermolecular interactions between the local reactants have been identified by taking into account the reduced density gradient obtained after a single point calculation performed via the PBE [8] exchange and correlation functional. This kind of calculation has been conducted by following the standard procedure [7] and therefore by selecting a correct isosurface in order to map the real space regions where the NCI act. As it is clear in both cases, strong NCI arise when the two counterions are close to each other (as it can be argued also from the previous Löwdin population analysis). This means that a non-negligible force acts in both gas phase systems just in the portion of space which lies in the middle of the two counterions. This *neighbouring effect*, as confirmed also by some standard electron density calculations (not shown here), perturbs the respective molecular orbitals. This represents the reason that underlies the evidence, emerged both through Car-Parrinello [9] and Born-Oppenheimer molecular dynamics simulations in the gas phase starting from these two atomic configurations, that both reactions proceed spontaneously even without the external electric field. This way, in liquid, the reaction pathways (*i.e.*, the intermediate and transition states) would appear to be dramatically different from any

thinkable gas phase counterpart of the reactions here presented.

---

[1] Pietrucci, F. & Saitta, A. M. Formamide reaction network in gas phase and solution via a unified theoretical approach: Toward a reconciliation of different prebiotic scenarios. *Proc. Natl. Acad. Sci. USA* **112**, 15030-15035 (2015).

[2] Cassone, G., Pietrucci, F., Saija, F., Guyot, F. & Saitta, A. M. One-step electric-field driven methane and formaldehyde synthesis from liquid methanol. *Chemical Science* **8**, 2329-2336 (2017).

[3] Marzari, N. & Vanderbilt, D. Maximally localized generalized Wannier functions for composite energy bands. *Phys. Rev. B* **56**, 12847-12865 (1997).

[4] Marzari, N., Mostofi, A. A., Yates, J. R., Souza, I. & Vanderbilt, D. Maximally localized Wannier functions: Theory and applications. *Rev. Mod. Phys.* **84**, 1419-1475 (2012).

[5] Löwdin, P.-O. On the nonorthogonality problem. *Adv. Quantum Chem.* **5**, 185-199 (1970).

[6] Bolhuis, P. G., Chandler, D., Dellago, C. & Geissler, P. L. Transition path sampling: throwing ropes over rough mountain passes, in the dark. *Annual Rev. of Phys. Chem.* **53**, 291-318 (2002).

[7] Contreras-García, J. *et al.* NCIPLOT: a program for plotting noncovalent interaction regions. *J. Chem. Theo. Comp.* **7**, 625-632 (2012).

[8] Perdew, J. P., Burke, K. & Ernzerhof, M. Generalized gradient approximation made simple. *Phys. Rev. Lett.* **77**, 3865 (1996) and *Phys. Rev. Lett.* **78**, 1396 (1997).

[9] Car, R. & Parrinello, M. Unified approach for molecular dynamics and Density-Functional Theory. *Phys. Rev. Lett.* **55**, 2471 (1985).
